# The Expanding Role of GLP-1 Receptor Agonists: Advancing Clinical Outcomes in Metabolic and Mental Health

**DOI:** 10.3390/cimb47040285

**Published:** 2025-04-17

**Authors:** Mohamad Al Qassab, Mohammad Mneimneh, Ahmad Jradi, Bassem Derbas, Dana Dabboussi, Justine Khoury Baini, Nadia Katrib, Nadim Chaarani, Philippe Attieh, Amjad Kanaan, Frederic Harb, Sami Azar, Hilda E. Ghadieh

**Affiliations:** Department of Biomedical Sciences, Faculty of Medicine and Medical Sciences, University of Balamand, Al-Koura, Tripoli P.O. Box 100, Lebanon; mohamad.alqassab@std.balamand.edu.lb (M.A.Q.); mohammad.mneimneh@std.balamand.edu.lb (M.M.); ahmad.jradi@std.balamand.edu.lb (A.J.); bassem.derbas@std.balamand.edu.lb (B.D.); dana.dabboussi@std.balamand.edu.lb (D.D.); justine.khourybaini@std.balamand.edu.lb (J.K.B.); nadia.katrib@std.balamand.edu.lb (N.K.); nadim.chaarani@std.balamand.edu.lb (N.C.); philippe.attieh@std.balamand.edu.lb (P.A.); amjad.kanaan@balamand.edu.lb (A.K.); frederic.harb@balamand.edu.lb (F.H.); sami.azar@balamand.edu.lb (S.A.)

**Keywords:** Glucagon-like peptide 1 receptor agonists, metabolic health, type 2 diabetes mellitus, diabetic nephropathy, fatty liver disease, cardiovascular diseases, neuropsychiatric disorders, Alzheimer’s disease, Parkinson’s disease, dementia, genetic polymorphisms

## Abstract

Glucagon-like peptide 1 receptor agonists (GLP-1 RAs) have emerged as a promising therapeutic option beyond their established role in managing type 2 diabetes mellitus (T2DM) and obesity. Recent research has highlighted their beneficial effects on liver, kidney, and cardiovascular health, mediated by both direct and indirect mechanisms. In the liver, GLP-1 RAs contribute to the improvement of metabolic dysfunction-associated steatotic liver disease (MASLD) by reducing hepatic fat accumulation, inflammation, and oxidative stress. Additionally, they enhance insulin sensitivity and lipid metabolism. Similarly, in diabetic kidney disease (DKD), GLP-1 RAs exhibit renoprotective properties by mitigating inflammation, oxidative stress, and glomerular hypertension. Furthermore, they promote natriuresis and stabilize renal function. Moreover, GLP-1 RAs present significant cardiovascular benefits, including improved myocardial function, reduced atherosclerosis progression, enhanced endothelial health, and decreased major adverse cardiovascular events (MACEs). Additionally, emerging evidence suggests GLP-1 RAs may exert substantial neuropsychiatric benefits, including reductions in depressive symptoms, anxiety, substance use behaviors, and lowering the risk of Alzheimer’s disease, Parkinson’s disease, and other dementias likely mediated by the modulation of neurotransmitter systems and neuroinflammation. Genetic polymorphisms in the GLP1R gene also impact the therapeutic response, highlighting the importance of personalized medicine in optimizing GLP-1 RA efficacy. This review synthesizes preclinical and clinical evidence supporting the multifaceted effects of GLP-1 RAs across multiple organ systems, highlighting their therapeutic potential beyond glycemic control. As research advances, further exploration of their mechanisms of action and long-term clinical outcomes, safety and effectiveness across diverse patient populations will be essential in optimizing their use in treating metabolic and neuropsychiatric conditions.

## 1. Introduction

Glucagon-like peptide 1 receptor agonists (GLP-1 RAs) have become indispensable for both patients and clinicians in managing type 2 diabetes mellitus (T2DM) and obesity. The advantages of GLP-1 RAs stem from their multifaceted effects, including glucose-dependent insulin secretion, inhibition of glucagon secretion, glycemic regulation, and slowed gastric emptying [1]. The GLP-1 RAs endogenously act like the incretin hormone glucagon-like peptide 1 (GLP-1) which increases postprandially [2]. GLP-1 is a 30-amino acid intestinal peptide hormone produced and secreted by enteroendocrine L cells, whereas GIP is a 42-amino acid peptide produced by enteroendocrine K cells of the upper intestine which both interact with insulin secretion after food intake, maintaining glucose homeostasis postprandially [3,4,5,6,7]. The surge in GLP-1 stimulates the synthesis of insulin which inhibits glucagon release, leading to the delay in gastric emptying. The delay aids in decreasing the postprandial serum glucose levels and suppressing appetite, and leads to weight loss. GLP-1 RAs are categorized into two main classes based on their molecular structure: those derived from the human GLP-1 backbone and those derived from the exendin-4 backbone. The human GLP-1 backbone class comprises dulaglutide, albiglutide, liraglutide, and semaglutide. On the other hand, the exendin-4 backbone class includes exenatide (available in two formulations) and lixisenatide [8]. All the formulations of the GLP-1 RA are administered subcutaneously due to the poor oral availability [8].

Receptors for both GIP and GLP-1 are not exclusive to the islets of Langerhans of the pancreas; instead, they are expressed throughout the body. For instance, GIP receptors are highly expressed in the testicles while GLP-1 receptors are extensively found in the lungs and duodenum, inferring that these two incretins have different physiological activities [9]. GLP-1 Rs are found in the terminal ileum, colon, both α and β cells in the pancreas, heart, lungs, and kidneys, and in several brain regions responsible for food intake and satiety [10]. To this day, the expression of GLP-1 Rs in hepatocytes is still a controversial topic. Using human hepatocyte biopsies or animal models, there is evidence that GLP receptors are present on hepatocytes, inferring direct effects of GLP-1 RAs. Collectively, GLP-1 RAs positively affect the dysregulation found in metabolic syndromes [3,11,12]. In the liver, GLP-1 RA agonists have shown significant improvement of hepatic steatosis by decreasing triglycerides [4,12] and fatty acid-induced endoplasmic reticulum (ER) stress and apoptosis, in addition to promoting autophagy of free fatty acids [11,13,14]. Patients with diabetic kidney disease (DKD) have enhanced renal outcomes with the use of GLP-RAs. The receptor agonists play a direct role in inflammation and oxidative stress reduction, as well as promotion of natriuresis. Additionally, they exert indirect effects by addressing factors like hyperglycemia, dyslipidemia, hypertension, and obesity [15]. GLP-1 RAs offer notable cardiovascular benefits, enhancing left ventricular ejection fraction, myocardial contractility, coronary blood flow, cardiac output, and endothelial function. They also diminish infarction size and mitigate the overall risk of cardiovascular events [8]. Moreover, recent evidence indicates that GLP-1 RAs may offer psychiatric benefits, particularly concerning mood disorders, anxiety, cognitive impairment, and substance use disorders. The intersection between metabolic health and mental health disorders further supports exploring these medications within psychiatry and addiction medicine [16]. Notably, recent evidence highlights a growing role in neurodegenerative diseases. GLP-1 RAs enhance insulin sensitivity in neurons, counteracting central insulin resistance—an emerging hallmark of Alzheimer’s disease (AD) and Parkinson’s disease (PD) [17,18]. They also attenuate amyloid-beta (Aβ) accumulation, tau phosphorylation, neuroinflammation, and oxidative stress, thereby offering neuroprotection and cognitive benefits [19,20].

Genetic polymorphisms within the GLP-1 receptor gene further impact these various therapeutic outcomes, highlighting the importance of personalized approaches in clinical treatment [21,22]. This review synthesizes current preclinical and clinical research to demonstrate the growing therapeutic profile of GLP-1 RAs, emphasizing their significant potential in treating multiple conditions associated with metabolic dysfunction and mental health.

## 2. Hepatoprotective Effects of GLP-1 RAs

### 2.1. Preclinical Evidence: GLP-1RAs in Liver Protection

Metabolic dysfunction-associated steatotic liver disease (MASLD) is the most common liver disease due to the wide spread of metabolic syndrome, obesity, and T2DM. The incidence rate of MASLD has been increasing and is currently estimated at around 46.13 per 1000 person-years. It is characterized by the presence of steatosis in no less than 5% of hepatocytes which can progress to inflammation and fibrosis leading to metabolic dysfunction-associated steatohepatitis (MASH) and liver cirrhosis [3,4,23,24]. Studies have shown that greater insulin resistance is associated with a higher risk of developing MASLD with ballooning and hepatic lobular inflammation [25]. The complex relationship between T2DM and MASLD is primarily mediated by insulin resistance. The risk of developing T2DM as a comorbidity of MASLD is doubled, and vice versa [26]. The two diseases have several important implications such as shared pathogenic mechanisms, mutual exacerbation, and cardiovascular risk. A vicious cycle of T2DM and insulin resistance leads to the accumulation of fat in the liver and MASLD, which further aggravates insulin resistance and T2DM [27]. The relationship between insulin resistance and liver fat production leads to MASLD being a comorbidity of T2DM. In T2DM, poor glycemic control leads to increased insulin resistance, which in turn promotes hepatic fat accumulation. These conditions can progress to a more severe form of MASLD, known as MASH; an advanced liver disease characterized by inflammation and fibrosis. Additionally, there is a markedly increased risk of cardiovascular disease, dyslipidemia, hypertension, carotid atherosclerosis, arterial calcification, and endothelial dysfunction [27]. Since MASLD and MASH are closely linked to T2DM, researchers have been investigating the efficacy of glucose-lowering incretins. Glucose-dependent insulinotropic polypeptide (GIP) and glucagon-like peptide 1 (GLP-1) are the two primary incretin hormones secreted by the intestine in response to glucose or nutrient ingestion, stimulating insulin secretion from pancreatic β cells [28,29].

The potentially beneficial effects of GLP-1 RAs (Table 1) range from promoting lower postprandial glucose levels, to increasing insulin and suppressing glucagon secretions, and better glycemic control, all of which are critical in managing MASLD. GLP-1 RAs help decrease appetite by decreasing serotonin 5-Hydroxytryptamine (serotonin) 2A (5-HT2A) receptors in the hypothalamus and inhibiting the activity of neuropeptide Y (NPY) and agouti-related protein (AgRP) neurons in the arcuate nucleus. This slows gastric emptying, leading to a more gradual rise in blood glucose and maintaining postprandial glucose homeostasis. The early satiety feeling that ensues leads to lower food intake, weight loss, decreased insulin resistance, and the improvement of MASLD. The reduction in hepatic lipid accumulation and inflammation deters the progression into MASH [3,4,28,30,31,32,33,34,35]. Total body weight loss is directly linked with decreasing hepatic fat content. A decrease in the total weight of 5% is associated with a 10% decrease in overall liver volume and visceral adipocytes, whereas hepatic triglyceride content is reduced by 40%. Moreover, weight loss improves insulin sensitivity and clearance rates, which positively impacts liver health [28,36,37,38].

Abnormal hepatic glucose and lipid homeostasis lead to hepatic steatosis; the hallmark of MASLD. Treatment with GLP-1 RAs has shown significant improvement in hepatic steatosis by inhibiting cell death and stimulating lipolysis [4,39]. Using Huh7 and HepG2 cell culture models, the GLP-1 RA liraglutide decreased triglyceride accumulation and the expression of sterol regulatory element binding protein 1c (SREBP-1c) and stearoyl-CoA desaturase-1 (SCD-1) involved in lipogenesis, but increased peroxisome proliferator-activated receptor α (PPAR-α), enhancing β-oxidation of free fatty acids (FFAs) and the signaling cascade downstream of the insulin receptor [40,41]. Similar effects were seen in human hepatocytes where GLP-1 RAs decreased fatty acid-induced endoplasmic reticulum (ER) stress, apoptosis, and promoted autophagy of FFAs and lipid degradation by the transcription factor EB (TFEB)-mediated autophagy–lysosomal pathway [11,13,14,38]. The decrease in ER-stress, apoptosis, and intrahepatic accumulation of FFAs can prevent the progression of MASLD into MASH [3,42]. The reduction in lipid accumulation by GLP-1 RAs may be a result of alterations in synthesis transport or clearance. For example, liraglutide favors reverse cholesterol transport whereby cholesterol is transported to liver for elimination as bile salts [43,44]. Moreover, GLPA-1 RAs are effective in decreasing hepatic de novo lipogenesis by reducing the activation of carbohydrate-responsive element-binding protein (ChREBP), a modulator of liver lipogenesis [34,45]. They also decreased hepatic very-low-density lipoprotein (VLDL) production by reducing the expression of SREBP-1c and fatty acid synthase (FASN) [34,46]. In addition to increased lipid accumulation, chronic inflammation contributes to the development of MASH. As such, the liver exhibits elevated levels of inflammatory cytokines and activated inflammasome in Kupffer cells (KCs). Liraglutide lowered NLR family pyrin domain containing 3 (NLRP3) inflammasome activation in KCs and decreased mitochondrial dysfunction, leading to an important reduction in IL-1β, IL-12, and tumor necrosis factor-α (TNF-α) expression levels, while increasing the expression of IL-10 [47,48].

Elevated fat accumulation levels lead to increased inflammation in the liver, promoting the release of profibrogenic factors and proinflammatory cytokines that contribute to fibrosis [49,50,51,52]. CEACAM1 promotes insulin sensitivity by enhancing insulin clearance and reducing liver fat production. Mice lacking CEACAM1 (*Cc1−/−)* develop hyperinsulinemia, insulin resistance, and steatohepatitis, along with early fibrosis [49,53]. Ghadieh et al. used an animal model where mice deficient in CEACAM1 (*Cc1−/−*), having spontaneous fibrosis, were fed with a high-fat diet, just to progress into a severe bridging fibrosis resembling the human MASH [49]. In this study, exenatide induces Ceacam1 expression and improves insulin clearance, insulin resistance, and liver fat accumulation in high-fat diet-fed mice. It also restores liver enzyme activities and reduces inflammation, oxidative stress, and pro-fibrogenic pathways in a CEACAM1-dependent manner. These findings suggest that exenatide prevents liver injury and fibrosis by promoting CEACAM1 activity [49]. In another study, exenatide was shown to upregulate CEACAM1 expression in high-fat diet-fed mice. This regulation was linked to improved insulin sensitivity and reduced hepatic steatosis. Exenatide promoted Ceacam1 expression through PPARγ binding to its promoter, and this effect was blocked by a GLP-1 receptor antagonist [38]. The findings highlight the importance of insulin metabolism in maintaining sensitivity and lipid balance, positioning exenatide as a potential therapeutic for metabolic disorders. In another study, liraglutide reduced inflammatory cytokines and decreased apoptosis and reactive oxygen species (ROS) levels, which resulted in the decreased activation of hepatic stellate cells, which is essential for the progression of MASH to fibrosis [3,54].

**Table 1:** Summary of GLP-1 RA mechanisms and their impact on MASLD/MASH.

Mechanism	Description	Impact on the Progression of MASLD/MASH	Ref.
Insulin Sensitivity and Glucagon Suppression	GLP-1RAs enhance insulin secretion and suppress glucagon release, improving insulin sensitivity.	Reduces MASLD risk by addressing insulin resistance.	[4,39]
Gastric Emptying and Appetite Regulation	Slows gastric emptying, regulates gastrointestinal movement, and induces satiety, lowering postprandial glucose levels.	Indirectly mitigates MASLD risk through weight reduction.	[34,35]
Decrease in Hepatic Fat Accumulation	Demonstrated efficacy in reducing liver fat through animal models and possibly direct effects on human hepatocytes.	Prevents MASLD/MASH development by reducing hepatic steatosis.	[4,12,34,40,55,56]
Lipogenesis and Modulation of Fatty Acid Oxidation	Reduces expression of genes related to de novo lipogenesis and enhances β-oxidation of free fatty acids.	Improves hepatic steatosis by altering lipid metabolism.	[34,45]
Oxidative Stress and Inflammation Reduction	Reduces oxidative stress and inflammation, crucial in MASLD progression.	Prevents or delays liver damage by diminishing hepatocyte degeneration and inflammation.	[3,42]
Cholesterol Transport and Lipid Accumulation	Facilitates cholesterol transportation to the liver for elimination, reducing lipid accumulation.	Aids in preventing liver injuries by enhancing reverse cholesterol transport.	[43,44]
Inflammatory Cytokines and Inflammasome Activity	GLP-1RAs like liraglutide have shown to decrease NLRP3 inflammasome activation and reduce inflammatory cytokines.	Prevents the progression of MASLD into MASH by modulating inflammatory responses.	[47]
Fibrosis Resolution	Exenatide restores CEACAM1 expression, reducing fat accumulation, inflammation, and fibrosis progression.	Reverses fibrosis progression in MASH by inhibiting oxidative stress, inflammatory pathways and reducing collagen deposition.	[49,50,51,54]

Abbreviations: Glucagon-like peptide 1 receptor agonists (GLP-1 RAs), metabolic dysfunction-associated steatotic liver disease (MASLD), metabolic dysfunction-associated steatohepatitis (MASH), NLR family pyrin domain containing 3 (NLRP3), and carcinoembryonic antigen-related cell adhesion molecule 1 (CEACAM1).

### 2.2. Clinical Insights: GLP-1RAs in Liver Disease Management

Several trials have been conducted to evaluate the efficacy of GLP-1 RAs in the management of MASLD in patients with T2DM. Clinical guidelines for MASLD management target weight loss through diet and lifestyle modifications, without any pharmacotherapies approved for the treatment of MASLD [57]. In this section, we will discuss the therapeutic effects of GLP-1 RAs on hepatic health (Table 2) as observed in clinical trials. The main goal of GLP-1 RA use for MASH is decreasing liver fibrosis by promoting fat loss and improving insulin sensitivity. The triad of obesity, high body fat percentage, and insulin resistance leads to liver inflammation, which has been associated with hepatic fibrosis. The mechanism of liver fibrosis is believed to be due to the excess supply of non-esterified free fatty acids (NEFAs) to the liver, increased mitochondrial β-oxidation leading to an increase in reactive oxygen species (ROS) production, and subsequent inflammation and extracellular matrix (ECM) remodeling by hepatic stellate cells (HSCs) [58]. The reduction in liver fibrosis is often associated with a decrease in liver enzymes back towards pre-MASLD normal values. Elevated liver transaminases are an independent predictor of MASLD, and a substantial proportion of patients with T2DM and elevated transaminases have MASLD or MASH [59]. Armstrong et al. demonstrated that 39% of patients that were randomly assigned to receive liraglutide showed resolution of definite MASH when compared with only 9% of patients in the placebo group [60]. Concomitantly, the administration of lixisenatide in patients with elevated liver enzymes showed a normalization in ALT levels when compared with placebo or other glucose-lowering agents [59]. Similar results were echoed in the trial conducted by Shao et al. where exenatide was superior to insulin therapy in producing hepatic-protective effects by decreasing liver function enzymes, fasting blood glucose, postprandial blood glucose, HbA1c, total bilirubin levels, total cholesterol, and triglyceride [61].

Many pharmacotherapies for T2DM, such as metformin therapy, have not shown a significant histological effect on MASLD [57,62]. Yan et al. evaluated the efficacy of liraglutide, sitagliptin, and insulin glargine in patients with T2DM and MASLD with inadequate glycemic control [55]. Liraglutide treatment significantly reduced liver fat content, whereas insulin glargine failed to produce the same effect. Another GLP-1 RA, dulaglutide, showed similar findings, where the administration of this drug showed an absolute decrease of 26.4% in liver fat content [63]. Furthermore, GLP-1 RAs, like semaglutide at a certain dose, are associated with a significant improvement in MASH resolution with no significant improvement in fibrosis progression, as seen in a phase 2 clinical trial conducted by Newsome et al. [64]. A meta-analysis of 615 patients assessed the effects of GLP-1 RA treatment on liver health in individuals with T2DM. Over a follow-up period of up to 72 weeks, GLP-1 RA significantly reduced liver enzymes, liver fat content, triglycerides, and HbA1c compared to standard care, indicating improved hepatic function and reduced inflammation. Biopsy results also showed notable liver disease resolution. These findings support GLP-1 RA as a promising treatment option for metabolic-associated liver disease when combined with lifestyle modifications [65]. A phase 2 clinical trial by Newsome et al. [64] evaluated the efficacy and safety of semaglutide in patients with MASH and liver fibrosis. Over 72 weeks, 320 patients received different doses of semaglutide or a placebo. The highest dose (0.4 mg) led to a significantly greater resolution of MASH without worsening fibrosis compared to placebo. However, no significant difference was observed in fibrosis improvement between the groups. These results suggest that semaglutide effectively improves MASH but may not directly impact fibrosis progression [64].

**Table 2:** GLP-1 RAs approved for clinical use and their effects on liver function.

Drug Name	Brand Name	FDA Approval Date	Outcome	Ref.
Semaglutide	Ozempic (injection), Rybelsus (OA)	2017, 2019	Resolution of steatohepatitis and no worsening of liver fibrosis. Improvement of liver fibrosis and no worsening of steatohepatitis. Decrease in MASLD activity score.Reduction in liver fat content and reduction in ALT.	[64,65]
Lixisenatide	Adlyxin	2016	Normalization of ALT.	[59]
Semaglutide + Empagliflozin	Ozempic + Jardiance	N/A	Histological resolution of MASH without worsening fibrosis.	[34,64]
Cotadutide	N/A	N/A	Resolution of MASH without worsening of liver fibrosis.	[66]
Exenatide	Byetta and Bydureon	2012	Decreasing liver function enzymes, fasting blood glucose, postprandial blood glucose, HbA1c, total cholesterol, triglyceride, and total bilirubin levels.	[61]
Liraglutide	Victoza	2010	Resolution of definite MASH.	[55,60]
Tirzepatide	Mounjaro	2023	Change in liver fat quantification.	[67]
Dulaglutide	Trulicity	2024	Decreased liver fat content.	[63]

Abbreviations: Alanine transaminase (ALT), metabolic dysfunction-associated steatotic liver disease (MASLD), and metabolic dysfunction-associated steatohepatitis (MASH). N/A: not available.

## 3. Renoprotective Effects of GLP-1 RAs

### 3.1. Preclinical Evidence: GLP-1RAs in Kidney Protection

The primary cause of mortality and morbidity in T2DM is diabetic kidney disease (DKD) [68]. GLP-1RAs have been shown to improve the renal outcomes of DKD either directly, by reducing the inflammation and oxidative stress and promoting natriuresis, or indirectly, by managing hyperglycemia, dyslipidemia, hypertension, and obesity [15].

T2DM causes a chronic low-grade inflammation in the kidneys [15]. This induces a rise in the systemic oxidative stress that worsens the staging of the developing DKD [68]. GLP1-RAs mitigate the impact of T2DM on the progression of DKD by suppressing many of the inflammatory signaling pathways like the renal nicotinamide adenine dinucleotide phosphate (NADPH) oxidase [15,68]. This anti-inflammatory effect occurs via the inhibition of protein kinase C (PKC) and the activation protein kinase A (PKA), leading to an increased level of cAMP [69]. This will reduce the production of adhesion molecules and proinflammatory cytokines, hence improving microalbuminuria and the histological changes seen in DKD [15]. The proliferation of the human mesangial cells and fibrosis were suppressed upon increased PKA activation and cAMP levels following exendin-4 administration [70]. Moreover, GLP1-RAs have been shown to downregulate the hyperglycemia-induced expression of nuclear factor kappa B (NF-κB) that is a major mediator of renal inflammation [71]. Decreased levels of NF-κB inhibit TNF-α expression in the glomerular podocytes and increases the levels of eNOS in endothelial cells [15]. Giving liraglutide to obese mice with glomerulonephropathy reduced podocyte damage by a TNF-α-mediated NF-κB activation [72]. In addition, overexpression of GLP-1 in diabetic mouse models (*db/db)* has shown to decrease the transcription of TNF-α and CCL5, with a subsequent decline in CD3+ T cells and F4/80+ macrophage infiltration into the kidneys [73]. Additionally, GLP1-RAs reduce inflammation through reducing oxidative stress. Exendin-4 limits the oxidative stress by activating the antioxidative Nrf2-mediated signaling pathway [74,75], whereas liraglutide decreased the concentration of heme oxygenase-1 and lipid hydroperoxides in the serum while simultaneously increasing glutathione levels [76].

GLP1-RAs promote natriuresis and urine flow in both healthy individuals and those with T2DM [77]. PKA activated by these agonists phosphorylates the sodium–hydrogen exchanger 3 (NHE3), a channel located on the brush border of the proximal tubule epithelial cells, at sites Ser552 and Ser605, leading to its inhibition [78]. The resulting decrease in the activity of this channel activates tubuloglomerular feedback by increasing sodium delivery to the macula densa [68]. This will further alter the glomerular hemodynamics by causing vasoconstriction of the afferent arteriole, hence decreasing the hyperfiltration and the pressure inside the glomerulus [78]. This effect is seen with liraglutide, which initially causes an abrupt decrease in the glomerular filtration rate (eGFR) and its stabilization afterward [68]. GLP1-RA infusions reduce sodium reabsorption at the proximal tubule by decreasing renin and angiotensin II levels [79]. Moreover, only one subcutaneous injection of liraglutide increases the excretion of sodium in T2DM individuals [80]. Additionally, GLP1-RAs modulate the concentration of calcium, chloride, and potassium in the tubules by interfering with their tubular exchange, leading to an enhanced natriuresis and diuresis [15]. In rats, this natriuresis is further promoted by an increase in renal blood flow after GLP1-RA administration [81].

In addition to their direct effects, GLP1-receptor agonists indirectly improve outcomes in DKD by lowering blood pressure, enhancing glycemic control, and reducing obesity and dyslipidemia. Obesity, a significant global health burden, is strongly associated with cardiovascular diseases, strokes, depression, infertility, and other complications. The influence of GLP-1 on obesity has played a key role in its clinical relevance. GLP-1 receptors are expressed in various brain regions involved in regulating appetite [82]. Unlike native GLP-1, GLP-1 agonists possess an extended half-life, enabling sustained activity, thus facilitating weight loss [83]. Furthermore, GLP1-RAs slow gastric emptying, which enhances gut distention, the rate of nutrient absorption, and modulates the secretion of appetite-regulating gut hormones. Moreover, the delayed gastric emptying also contributes to lowering postprandial glucose levels [84]. This effect is relevant given the kidney’s role in blood glucose regulation through processes like gluconeogenesis and glomerular filtration [85]. Impaired kidney function is common in diabetes with an average of 40% of patients with T2DM and 30% with T1DM that have been noticed to have a low glomerular filtration rate. On a related note, GLP1-RAs have been shown to mitigate the decline in estimated eGFR by increasing insulin secretion and decreasing glucagon secretion. This dual hormonal regulation is crucial for lowering glucose levels, particularly in cases where kidney function is preserved [85]. Additional indirect renal benefits of GLP-1 RAs include reductions in blood pressure and dyslipidemia, further supporting their role in mitigating diabetic kidney disease progression.

### 3.2. Clinical Insights: GLP-1RAs in Renal Disease Management

Patients with T2DM are at a higher risk for the development and progression of DKD. Approximately 50% of people with T2DM will develop DKD in their lifetime, and approximately half of kidney failure cases are attributed to diabetes. In patients with T2DM, the use of GLP-1 RAs has been correlated with kidney-protective effects (Table 3) which were more significant in patients with pre-existing chronic kidney disease. Data were taken from the SUSTAIN 6 (Trial to Evaluate Cardiovascular and Other Long-Term Outcomes with Semaglutide in Subjects with T2DM; n = 3297) and LEADER (Liraglutide Effect and Action in Diabetes: Evaluation of Cardiovascular Outcome Results; n = 9340) trials to assess albuminuria and eGFR reduction from baseline. In a 2.1-year median for follow up for SUSTAIN 6 and a 3.8-year median for follow up for LEADER, a statistically significant decrease in albuminuria was observed from baseline when a combination of semaglutide/liraglutide was used. The eGFR slope also saw a significant decline deceleration with semaglutide and liraglutide when compared to a placebo [86]. The nephroprotective effects of semaglutide and liraglutide were larger in patients with a lower baseline eGFR. Indeed, patients with an eGFR of 30 to 60 mL/min/1.73 m^2^ had a higher likelihood of persistent reduction for all thresholds [86]. There was a clear improvement of kidney function and overall kidney health reinforcing the efficacy of two commercially available GLP-1 RA drugs when used in patients with T2DM. The LEADER trial has shown that GLP1-RAs reduce systolic blood pressure by redistributing the activity of Na^+^/H^+^ present in the renal proximal tubules and decreasing plasma angiotensin II [82]. GLP-1 agonists are associated with a reduction in triglyceride, either by clearing lipids from circulation through the activation of brown adipose tissue or by impairing lipogenesis in the liver through the AMP-activated protein kinase [84].

GLP-1 RAs were also used in combination with another class of hyperglycemic medication: the sodium glucose cotransporter 2 (SGLT-2) inhibitors. In a clinical case report conducted by Kuhadiya et al., the addition of a SGLT-2 inhibitor alongside a GLP-1 RA in high-risk renal T2DM patients led to a slowing of the progression to end stage kidney disease and decreased morbidity from renal or cardiovascular causes [87]. In some cases, a triple therapy of angiotensin receptor blockers (ARBs), GLP-1 RA, and SGLT-2 inhibitor have been suggested to have a superior nephroprotective effect in patients suffering from advanced-stage nephropathy. GLP-1 RAs act on the glomerulus, preventing its hypertrophy and mesangial expansion, ARBs halt mesangial expansion and dilate the glomerular efferent arteriole, while SGLT-2 inhibitors reduce glucose reabsorption, reduce tubular oxidative stress, and reactivate the tubular glomerular feedback [88].

**Table 3:** Renoprotective effects of GLP-1 RAs.

Drug Name	Brand Name	Outcome	Ref.
Liraglutide	Victoza	Decreased proteinuria and rate of decline of GFR in T2DM with diabetic nephropathy.	[86]
Semaglutide	Ozempic	Decreased proteinuria and rate of decline of GFR.	[86]
Dulaglutide + SGLT-2 inhibitor	Trulicity	In T2DM patients with moderate to severe chronic kidney disease, eGFR is higher in those receiving GLP-1RAs compared to those on insulin therapy.	[87]
Lixisenatide	Adlyxin	No change.	[89]

Abbreviations: Estimated glomerular filtration rate (eGFR), type 2 diabetes mellitus (T2DM), sodium glucose cotransporter-2 (SGLT-2) inhibitors, and glucagon-like peptide 1 receptor agonists (GLP-1RAs).

## 4. Cardiovascular Benefits of GLP-1 RAs

### 4.1. Preclinical Evidence: GLP-1RAs in Cardiovascular Protection

GLP-1 RAs have been shown to be involved in several aspects of cardiac function and diseases. Their cardiovascular benefits include enhanced cardiomyocyte survival through the inhibition of apoptosis and improved total cardiac output following injury and heart failure [5]. Mammalian models, using GLP1-RAs, revealed improved left ventricular function after non-fatal cardiac events. This reduction in injury severity and infarct size may be explained by the increased myocardial cell glucose uptake in the presence of inhibiting reperfusion injury [90]. Reperfusion injury may, in part, be reduced by the known effects of GLP1-RAs on intracellular calcium ion modulation and homeostasis [91], which may account for the possible antiarrhythmic effects of GLP1-RAs [92]. The expression of GLP-1 receptors in the sinoatrial node further suggests a role for GLP1-RAs in heart rate regulation [5]. Moreover, a recent study described the expression of GLP-1 receptor mRNA in the ventricles of the heart; however, the localization of the protein is still unknown [93].

GLP1-RAs’ cardioprotective effects include the inhibition of atherosclerosis formation and progression [94]. GLP1-RAs play a crucial role in inhibiting the inflammation of the coronary arteries, protecting against endothelial dysfunction, reducing the risk of new plaque formation or rupture of pre-existing atheroma plaques [90]. Human GLP-1 RA proteins have been detected in the endothelial cells of the coronary artery [95] where they function as moderate regulators of endothelial-mediated contraction and vasodilation [90]. Furthermore, GLP1-RAs inhibit the expression of adhesion proteins VCAM-1 and ICAM-1 on the endothelial cell, limiting the binding of immune cells to the endothelium wall [90]. There are several GLP1-RA molecular mechanisms to reverse endothelial cell dysfunction. In addition to an immediate protective effect by the GLP-1R-dependent activated protein kinase/protein kinase B/endothelial nitric oxide synthase (AMPK/Akt/eNOS) pathway [96], GLP1-RAs enhance vascular wall integrity by protein kinase A (PKA) and Ras-related C3 botulinum toxin substrate 1 (Rac1) [90]. Furthermore, GLP-1 stabilizes endothelial contraction and acts as a barrier for AGE-treated endothelium by activating GLP-1R/cAMP/PKA and inhibiting mitogen-activated protein kinase (MAPK) signaling pathways [90].

In addition, GLP1-RAs have anti-inflammatory characteristics since they decrease TNFα and Interlukin-1 (IL-1) production and favor the transformation of macrophages from pro-inflammatory M1 macrophages to anti-inflammatory M2 macrophages, which secrete anti-inflammatory cytokines and promote tissue repair within the plaques [97]. Liraglutide hindered the effects of oxidized low-density lipoprotein (ox-LDL) by blocking the inhibitory impact of the p53 protein on kruppel-like factor 2 (KLF2), which plays a significant role in endothelial cell protection from ox-LDL in human aortic endothelial cells. Lixisenatide, another GLP-1 analogue, was shown to prevent cardiovascular attacks in apolipoprotein E−/− insulin receptor substrate 2+/− (*APOE−/−irs2+/−*) mice by narrowing the plaques, amplifying the stability of the plaque, and converting macrophages to anti-inflammatory M2 phenotype. Moreover, liraglutide uses the AMPK pathway to hinder the cell cycle of vascular smooth muscle cells, leading to a delay in atherosclerosis formation [1]. GLP-1 RAs have been found to inhibit the NF-kB pathway [95], which activates pro-inflammatory genes leading to the production of proinflammatory cytokines, such as TNFα and IL-6 [98]. Furthermore, GLP1-RAs have been reported to decrease systemic inflammation, therefore reducing C-Reactive protein (CRP) levels and thus CVD [97].

### 4.2. Clinical Insights: GLP-1RAs in Cardiovascular Outcomes

CVD is the leading cause of death in patients with T2DM, and patients with DKD have an elevated risk of cardiovascular complications in their lifetimes [99]. Therefore, it is important to find appropriate treatments and effective drugs to slow the progression of kidney disease, as well as reduce MACE through blood sugar and triglyceride control, body weight management, and normalizing blood pressure. Several trials (Table 4), with variable results, have been conducted looking for reduced CVD outcomes regardless of weight loss.

The results of a meta-analysis showed that baseline body mass index (BMI) was not associated with achieved glycemic control across seven different antihyperglycemic treatments [100]. Verma et al. also found that BMI does not affect MACE through weight and glucose level analysis [101]. In a more recent patient-level meta-analysis of eight cardiovascular outcome trials, Sattar et al. demonstrated that the cardiovascular and renal benefits of GLP-1 receptor agonists were consistent across all BMI categories, indicating that these effects are largely independent of baseline BMI [102]. Sattar et al. [102] found a 14% relative risk reduction in the 3-point MACE (*p* < 0.001). When analyzing the composite MACE components separately, GLP-1 receptor agonists were responsible for a reduction in cardiovascular mortality, fatal or non-fatal myocardial infarctions, and fatal or non-fatal strokes, as well as an 11% decreased risk for heart failure hospitalization and a 12% decreased risk of death from any cause (*p* < 0.001) [102]. However, the Harmony Outcomes trial (n = 10,793), which randomized patients with T2DM and high CVD risk to a drug regimen of the GLP-1 RA albiglutide or a placebo [103], noticed a significant reduction of 22% in the first occurrence of cardiovascular-related death, myocardial infarction or stroke [101]. The Researching Cardiovascular Events with a Weekly Incretin in Diabetes (REWIND) trial (n = 9901) also saw a significant decrease in cardiovascular events and body weight loss when dulaglutide was used versus placebo [104]. It is important to note that these seemingly contradictory findings are likely due to the sample size, location, and follow up with patients. In the meta-analysis conducted by Verma et al. [101], data were analyzed post hoc for the effects of liraglutide and semaglutide on the time to first MACE, cardiovascular-related death, and nephropathy, evaluated by baseline BMI. Their analysis showed no significance in the cardiovascular and renal benefits of two GLP-1 RAs versus placebo when analyzing the LEADER and SUSTAIN 6 trials. This discrepancy is explained by the difference in demographics examined for the effects of liraglutide and semaglutide. It was shown that these GLP-1 RAs showed consistent cardiovascular benefits across baseline BMI categories, mainly in T2DM patients already at high risk with CVD [101]. Similar results were found in the EXSCEL trial, where the incidence of MACE in patients with T2DM did not differ significantly between patients receiving exenatide and those receiving a placebo [105]. The ELIXA trial revealed similar findings, where patients taking lixisenatide did not show an improvement in MACE occurrence or rate of death [106]. In yet another clinical trial conducted by Marso et al. [107] (n = 3297), patients with T2DM who had high CVD risk reported a significantly lower rate of cardiovascular death, nonfatal myocardial infarction or nonfatal stroke while receiving semaglutide than those receiving a placebo. The study was limited by two main confounding factors: not being able to assess the efficacy for cardiovascular and renal outcomes across BMI groups, and the comparatively short follow up with patients [107]. Their analyses were also not adjusted for differences in insulin, SGLT-2 inhibitors, and cardiovascular medication use. These limitations were not able to exclude the possibility of effect modification by BMI for patients with T2DM taking GLP-1 RA. A case study regarding GLP1-RA effects on CVD in T2DM patients reported a strong positive correlation between the administration of GLP-1 RA and a reduced risk of CVD and MACE [108]. Many randomized control trials have shown that lifestyle changes and exercise are most efficient for CVD in T2DM patients [108]. However, GLP-1RAs have a significant effect in reducing CVD, including non-fatal myocardial infarctions, non-fatal strokes, and death from cardiovascular causes in T2DM patients [108]. Thus, the combination of life style modifications with GLP1-RA therapy may constitute a reasonable approach [108].

In addition to GLP-1 RA, the GLP-1 RA/SGLT-2 combination showed promising effects in the management of T2DM. In the systematic review and meta-analysis conducted by Mantsiou et al. [109], seven trials (n = 1913) were analyzed, and the combination therapy of GLP-1 RA and SGLT-2 inhibitors was associated with a greater reduction in HbA1c, body weight, and systolic blood pressure when compared to monotherapy using hyperglycemic drugs. However, data for mortality and cardiovascular outcomes using combination therapy were scarce [109]. It is imperative to keep in mind that further research and data collection are required to reach a final verdict on the effect of GLP-1 RA on cardiovascular health. The main cardioprotective effects of these hyperglycemic drugs are through anti-inflammatory pathways, lipid synthesis regulation, and the attenuation of cardiac ischemic injuries through interactions with the myocardium and coronary arteries by improving endothelial function [110]. In a meta-analysis conducted by Kristensen et al. [111], seven trials were included, involving over 56,000 participants, comparing the effects of different GLP-1 RAs, ELIXA (lixisenatide), LEADER (liraglutide), SUSTAIN6 (semaglutide), EXSCEL (exenatide), HARMONY (albiglutide), REWIND (dulaglutide), and PIONEER (oral semaglutide), against placebo. The findings indicated that GLP-1 RAs reduced major adverse cardiovascular events (MACEs) by 12%. Additionally, reductions were observed in cardiovascular death, stroke, and myocardial infarction. GLP-1 RAs also reduced all-cause mortality by 12%, hospital admissions for heart failure by 9%, and kidney-related outcomes by 17%, primarily through a reduction in urinary albumin excretion. Furthermore, no increase in the risks of severe hypoglycemia, pancreatitis, or pancreatic cancer was observed [111].

**Table 4:** Summary of clinical trials demonstrating the cardioprotective effects of GLP-1 RAs.

Trial	Prior CVD	Mean Follow Up (Months)	Outcome	Ref.
ELIXA (lixisenatide)	100%	25	No improvement in MACE or mortality rate.	[106]
HARMONY (albiglutide)	100%	18	Decreased risk of MI. No change in stroke risk, death from CV events or all-cause mortality.	[103]
SUSTAIN6 (semaglutide)	83%	26	Decreased risk of non-fatal stroke.	[107]
LEADER (liraglutide)	81%	46	Fewer occurrences of death due to MI.	[82]
EXSCEL (exenatide)	73%	38	No change in death from CV events, MI, stroke, HF hospitalizations or ACS hospitalizations.	[105]
REWIND (dulaglutide)	31%	65	Decrease in CV events and body weight loss.	[104]

Abbreviations: Major adverse cardiovascular event (MACE), myocardial infarction (MI), cardiovascular (CV), heart failure (HF), and acute coronary syndrome (ACS).

## 5. The Impact of GLP-1 RAs on Mental Health and Substance Use Disorders

### 5.1. Neurobiological Mechanisms Underlying Psychiatric Effects

GLP-1 receptors exhibit widespread expression in critical regions of the CNS, including areas involved in mood regulation, anxiety, reward processing, and cognition, notably like the hippocampus, amygdala, prefrontal cortex, hypothalamus, ventral tegmental area (VTA), nucleus accumbens (NAc), and nucleus tractus solitarius (NTS) [112,113]. The activation of these receptors has shown extensive neuromodulatory effects relevant to psychiatric conditions. The activation of GLP-1 receptors influences dopaminergic, serotonergic, and noradrenergic neurotransmitter systems. GLP-1 RAs specifically decrease dopamine release in the mesolimbic reward circuitry, thereby diminishing reward responses linked to addictive substances. Moreover, improvements in serotonin signaling related to enhanced insulin sensitivity may underline antidepressant effects observed with these agents [16,114]. Furthermore, GLP-1 RAs have potent anti-inflammatory effects in the brain, largely by suppressing inflammatory cytokines and inhibiting microglial activation [16,115,116]. Such anti-inflammatory activities are crucial since chronic neuroinflammation is firmly linked to the causes of depression, anxiety, and cognitive dysfunction. By reducing neuroinflammation and oxidative stress, GLP-1 RAs promote neuronal health and plasticity, thus enhancing cognitive and emotional resilience [16]. GLP-1 RAs also promote neurogenesis and neuroplasticity, principally by increasing brain-derived neurotrophic factor (BDNF) synthesis and signaling. Enhanced BDNF signaling contributes to neuronal development, synaptic plasticity, and enhanced resilience against stress-induced damage, which are key aspects in the therapeutic effects observed in mood disorders and cognitive impairment [16]. Finally, the normalization of stress responses mediated by the modulation of the hypothalamic–pituitary–adrenal (HPA) axis may also contribute to psychiatric benefits. Dysregulation of the HPA axis is a well-established pathophysiological mechanism underlying mood and anxiety disorders, and GLP-1 RAs may restore normal cortisol and stress hormone levels, leading to psychiatric stability [112,117,118,119].

### 5.2. Effects of GLP-1 RAs on Mental Health Disorders

#### 5.2.1. Depression

Preclinical research consistently shows that GLP-1 RAs possess antidepressant properties, significantly reducing depressive-like behaviors induced by chronic stress and inflammation in animal models. Dulaglutide and exenatide, in particular, demonstrated substantial antidepressant activity, correcting stress-induced behavioral alterations and lowering pro-inflammatory cytokines [16,120,121]. Clinical trials using GLP-1 RAs have yielded promising results, with multiple studies finding improved depression symptoms in patients with comorbid diabetes and obesity.

#### 5.2.2. Anxiety

Evidence for anxiolytic benefits of GLP-1 RAs has been accumulated, particularly in preclinical studies. Animal models treated with dulaglutide and exenatide showed reductions in anxiety behaviors as well as improvements in anxiety-related brain markers. The key mechanisms postulated are anti-inflammatory effects, enhanced neuroplasticity, and modulation of neurotransmitters involved in anxiety regulation, such as serotonin and GABA [16,121]. Although clinical data remains more limited, early human studies suggest beneficial reductions in anxiety symptoms among patients receiving GLP-1 therapy for metabolic indications.

#### 5.2.3. Suicidal Ideation and Self-Injury

Recent concerns about the safety of GLP-1 RAs emerged following pharmacovigilance reports indicating potential associations between liraglutide and semaglutide use and increased suicidal ideation and self-injury (SIS) [122]. However, rigorous analyses using large-scale cohort studies provided contrasting findings. A population-based propensity-weighted study involving 3040 patients initiating GLP-1 RAs found no significant increase in the incidence of SIS compared to alternative treatments, with hazard ratios of 1.04 (95% CI: 0.35–3.14) in per protocol and 1.36 (95% CI: 0.51–3.61) in intention-to-treat analyses, suggesting that the observed psychiatric side-effects might be confounded by other factors such as the psychological impact of rapid weight loss, unmet patient expectations, or underlying psychiatric comorbidities [117,122].

### 5.3. Effects of GLP-1 RAs on Alcohol and Nicotine Use Disorders

Clinical and preclinical evidence suggests that GLP-1 RAs significantly reduce alcohol and nicotine consumption. In a systematic review of clinical trials involving 630 participants conducted by Martinelli et al., they reported that three out of five studies demonstrated significant reductions in substance use, primarily alcohol and nicotine, compared to placebo. For example, dulaglutide and exenatide showed effectiveness in reducing alcohol intake and preference in human subjects [123]. Preclinical studies suggest these effects occur via the suppression of alcohol-induced dopamine release within the mesolimbic reward pathway, and decreased cue-induced craving responses, thus reducing the reinforcing properties of alcohol consumption [112,123,124].

### 5.4. Effects of GLP-1 RAs on Dementia

GLP-1 RAs improve insulin signaling in neurons, which is especially relevant as insulin resistance is increasingly accepted as a shared pathological feature of both Alzheimer’s disease and Parkinson’s disease, with some researchers even referring to AD as “type 3 diabetes” [18,125]. Insulin resistance suppresses neuronal glucose metabolism and uptake, disrupts synaptic plasticity, and facilitates the accumulation of neurotoxic proteins such as Aβ and hyperphosphorylated tau. GLP-1 RAs can reverse these effects by enhancing insulin sensitivity and activating neurotrophic signaling pathways such as the PI3K/Akt and MAPK/ERK cascades, promoting neuronal survival and synaptic remodeling [125,126].

Furthermore, GLP-1 RAs reduce oxidative stress and neuroinflammation—key mediators of neurodegeneration—through the regulation of microglial activation and suppressing proinflammatory cytokine production [17]. They also promote mitochondrial integrity and autophagy, which are essential to neuronal homeostasis [127]. In AD models, GLP-1 RAs have been shown to decrease Aβ deposition and tau phosphorylation while preserving synaptic density and cognitive function [125,128]. Exenatide and liraglutide have been shown in preclinical research to cross the blood–brain barrier and ameliorate AD-like pathology, with liraglutide improving cognition and reducing brain atrophy in animal models [128]. Clinically, a large propensity-matched cohort study involving over 5 million obese patients demonstrated that GLP-1 RA use significantly lowered the risk of developing AD (RR = 0.627), Lewy body dementia (RR = 0.590), and vascular dementia (RR = 0.438) [19]. Semaglutide, in particular, exhibited the highest neuroprotective efficacy, including a significant reduction in PD risk (RR = 0.574), highlighting its promise as a disease-modifying agent [19].

The neuroprotective benefits of GLP-1 RAs also extend to PD, where they appear to preserve dopaminergic neurons and enhance motor symptoms. Phase II clinical trials, using exenatide and liraglutide in PD patients, revealed that motor function was significantly enhanced compared to placebo, and ongoing phase III trials are assessing semaglutide’s efficacy [128]. A meta-analysis of 22 RCTs involving 138,282 participants reaffirmed that while most GLP-1 RAs showed no statistically significant prophylactic effect across the neurodegenerative spectrum, semaglutide was notably effective in reducing Parkinson’s incidence in subgroup analyses [129]. Importantly, treatment with GLP-1 RAs was shown to have a reduced hazard ratio for dementia in both RCT data (HR = 0.47, 95% CI: 0.25–0.86) and real-world evidence from a nationwide Danish cohort (HR = 0.89, 95% CI: 0.86–0.93), thereby supporting their potential for disease modification beyond metabolic control [130].

Despite these promising findings, some clinical studies report mixed results. A systematic review by Liang et al. found no significant cognitive improvements or reductions in Aβ/tau biomarkers in randomized trials of liraglutide and lixisenatide in AD patients [20]. Nonetheless, GLP-1 RAs showed metabolic benefits such as reduced BMI and improved cerebral glucose metabolism measured by ^18^F-FDG PET, suggesting they may preserve brain energy homeostasis—a hallmark in the prevention of neurodegeneration [20]. Complementing this, Yassine et al. highlighted that GLP-1 RAs can restore energy metabolism in the brain by improving glucose transport and mitochondrial function, which are often impaired in AD and related dementias [18].

## 6. Metabolic–Mental Axis and GLP-1 RAs

In addition to the well-characterized metabolic effects of GLP-1 RAs on organs such as the liver, kidneys, and heart, emerging evidence supports a putative link between metabolic regulation and cognitive function. This “metabolic–mental axis” highlights the bidirectional relationship between metabolic health and mental processes, including mood and cognition. Several studies have demonstrated that improvements in insulin sensitivity, glycemic control, and vascular function—hallmarks of GLP-1 RA action—may contribute to neuroprotection and enhanced cognitive performance [131,132].

GLP-1 RAs are capable of crossing the blood–brain barrier and have been shown to exert anti-inflammatory, anti-apoptotic, and neurotrophic effects within the central nervous system. These properties are particularly relevant in conditions such as Alzheimer’s disease and T2DM, where metabolic and neurocognitive dysfunction often coexist. For instance, liraglutide and semaglutide have demonstrated beneficial effects on learning and memory in both preclinical and clinical settings, possibly through improved insulin signaling and reduced oxidative stress in the brain [19,132,133].

Thus, integrating mental health considerations into the broader scope of metabolic disease management may offer a more holistic approach to treatment, especially in populations at risk for cognitive decline.

## 7. GLP-1R Gene Polymorphisms’ Influence on Responses to GLP-1 RAs Aiding Personalized Treatment in T2DM

GLP-1R is encoded by the GLP1R gene, located on chromosome 6p21.2. Various genetic changes in the GLP1R region, especially in the coding regions like exons and exon–intron junctions, as well as in regulatory regions such as the 5′ upstream regulatory regions, promoter, and 3′ untranslated regions, can contribute to genetic variability. Mutations in the GLP1R gene have been linked to altered GLP-1 receptor function, which can impact both insulin secretion from pancreatic cells after meals and the response to receptor agonists [21]. Several missense variants of GLP1R have been identified in patients with T2DM (Table 5).

### 7.1. Influence on Glycemic Response and Insulin Secretion in T2DM

A study conducted by Guan et al. aimed to investigate the association between genetic variants in the GLP1R gene and the therapeutic efficacy and gastrointestinal adverse drug reactions (ADRs) of GLP1-RAs in Chinese patients with T2DM. Adult T2DM patients were treated for 12 weeks with either exenatide or liraglutide. Genotyping of GLP1R rs10305420 and rs3765467 was conducted, and clinical responses were assessed based on changes in fasting plasma glucose (FPG), HbA1c, and body mass index (BMI). The study involved 176 participants, with 156 completing the treatment. Results showed that patients with the rs3765467 GG (wild type) genotype experienced a significantly greater reduction in HbA1c (1.7%) compared to those with the mutant (GA + AA) genotypes (0.8%). Additionally, the rate of achieving the target HbA1c of 7.0% was significantly higher in the GG genotype group (50.9%) compared to the GA + AA group (23.8%). However, gastrointestinal ADRs were similar across genotypes. The rs3765467 polymorphism in the GLP1R gene was then linked to a better therapeutic response to GLP1RAs, particularly in terms of HbA1c reduction, in Chinese T2DM patients [22].

Another study conducted by Eghbali et al. [134] analyzed data from a non-inferiority randomized clinical trial conducted between 2019 and 2020, where participants with T2DM received liraglutide (1.8 mg/day) for 24 weeks. Patients were stratified into four groups based on their baseline HbA1c levels: 7–7.99%, 8–8.99%, 9–9.99%, and ≥10%. Subjects who had an HbA1c reduction greater than the median for their group were classified as optimal responders. The study examined the genetic variants rs6923761 and rs10305420 of the GLP-1R gene using Sanger sequencing. Out of 233 participants, 120 were optimal responders, with a median HbA1c reduction of −2.5% in the optimal responder group compared to −1.0% in the suboptimal group (*p* < 0.001). The rs10305420 T allele homozygosity was associated with a better glycemic response to liraglutide in both recessive and codominant models. No significant association was found between the rs6923761 variant and HbA1c reduction. The rs10305420 polymorphism in the GLP-1R gene is then linked to a better glycemic response to liraglutide in Iranian patients with T2DM, with no response in the rs6923761 variant [134].

In terms of insulin secretion, it was shown that the two single nucleotide polymorphisms (SNPs) in the GLP1R gene, rs3765467 and rs10305492, can significantly reduce insulin secretion by β cells and lower cyclic AMP levels, while also promoting β cell apoptosis. Under high glucose conditions, both rs3765467 and rs10305492 similarly impaired insulin secretion and β cell viability. In other words, these variants in GLP1R affect glucose-stimulated insulin secretion in pancreatic β cells. Through leading to the dysfunction and apoptosis of β cells, GLP1R rs3765467 and rs10305492 might also impair GLP-1 interaction with GLP1R [135]. However, further research is needed to understand the exact mechanism by which these two SNPs can influence insulin secretion following GLP-1RA treatment in T2DM.

### 7.2. Influence on Stress Response in T2DM

GLP-1Rs are widely expressed in the brain. Evidence suggests that they may play a role in reward responses and neuroprotection. Yapici-Eser et al. [136] explored the association between GLP-1R polymorphisms and both objective and subjective measures of anhedonia, as well as depression diagnosis. The researchers assessed anhedonia through the Probabilistic Reward Task (PRT) and the Snaith–Hamilton Pleasure Scale (SHAPS), while also collecting clinical data and DNA samples from 100 controls and 164 patients. They used an independent sample from the Psychiatric Genomics Consortium (PGC) to examine the effect of GLP-1R polymorphisms on depression diagnosis. The results showed that the C allele of rs1042044 was significantly associated with increased response bias in the PRT, while the AA genotype of rs1042044 was linked to higher anhedonia scores based on SHAPS. However, no association was found between rs1042044 and depression diagnosis in the PGC data. The study suggests a potential link between rs1042044 and anhedonia but no association with depression diagnosis [136].

Stress in individuals with T2D can exacerbate the condition by triggering elevated blood glucose levels. Chronic stress and the HPA axis dysregulation can lead to insulin resistance and the worsening of metabolic parameters. Therefore, medications that influence stress response, like GLP-1RAs, can have indirect benefits for individuals with T2D by improving metabolic control and modulating the stress response. For instance, individuals carrying certain alleles of rs1042044 may exhibit either an enhanced or diminished response to GLP-1RAs, influencing both glucose regulation and the physiological stress response, with further research needed to confirm this potential association.

### 7.3. Influence on Cardiovascular Risk Factors in T2DM

As clinical trials have demonstrated a protective effect of GLP-1 RAs on the cardiovascular safety in patients with T2DM with high expression of GLP-1R in the cardiovascular system, several studies were conducted to examine if there are any potential associations between genetic variants of GLP-1R with cardiovascular complications among patients with T2DM [134]. A prospective study involving 104 Spanish treatment-naïve T2DM patients found that the GLP1R rs6923761 variant had no impact on anthropometric measurements, metabolic traits, or cardiovascular risk factors [137]. Furthermore, a study conducted by Melchiorsen et al. investigated 36 nonsynonymous GLP1R variants in 8642 Danish individuals to assess their impact on receptor signaling and cardiometabolic traits. Ten variants showed significantly reduced GLP-1-induced cAMP signaling, but no strong association with T2DM or major metabolic phenotypes was found. A broader analysis of predicted loss-of-function (pLoF) variants in 330,566 UK Biobank participants also revealed minimal cardiometabolic effects, aside from slight increases in fasting glucose and HbA1c. The absence of homozygous LoS or pLoF variants suggests evolutionary intolerance, highlighting GLP-1R’s potential importance in human physiology [138]. On the other hand, a study examining the impact of genetic variability on cardiovascular disease in T2DM patients from the Chinese Han population assessed the allele and genotype frequencies of 11 tag SNPs in the GLP1R region. In this study, which included 394 T2DM patients with coronary artery disease and 217 without, the rs4714210 GG genotype was found to be linked to a lower risk of cardiovascular disease. This association remained significant even after adjusting for other established cardiovascular risk factors [139]. The rs4714210 polymorphism in the GLP1R gene may influence how patients with T2DM respond to GLP-1RA therapy, particularly in terms of cardiovascular outcomes. While there is no direct clinical evidence linking rs4714210 to cardiovascular risk following GLP-1RA treatment, existing studies suggest that GLP-1RA therapies have significant cardiovascular benefits, and genetic variations like rs4714210 may modulate these effects. Further research is needed to confirm the exact role of rs4714210 in determining the efficacy of GLP-1RAs in reducing cardiovascular risk in T2DM patients.

### 7.4. Influence on Body Weight and Metabolic Parameters in T2DM

A study investigated the impact of the GLP1R rs6923761 polymorphism on metabolic changes and weight loss in 90 overweight Spanish T2DM patients who needed to start liraglutide treatment due to inadequate glycemic control with metformin alone. Following treatment with liraglutide, patients carrying the rs6923761 A allele experienced greater reductions in BMI, weight, and fat mass. However, there were no significant differences in the reduction in basal glucose levels, Homeostasis Model Assessment of Insulin Resistance (HOMA-R) index, or HbA1c between the two genotypes [140]. Chedid et al. [141] conducted a pilot study that examined the association of genetic variants in GLP1R (rs6923761) and TCF7L2 (rs7903146) with gastric emptying (GE) and weight loss in obese patients treated with liraglutide for 16 weeks or exenatide for 30 days. The results showed a significant correlation between weight changes and GE T1/2 (rs = −0.382, *p* = 0.004). GLP1R rs6923761 minor allele A carriers experienced greater delays in GE compared to GG genotype carriers. However, there was no significant difference in weight loss based on the GLP1R rs6923761 genotype after 5 weeks of treatment. No significant correlations were found with the TCF7L2 genotype [141]. Nevertheless, the impact of GLP1R rs6923761 on liraglutide’s ability to reduce weight was shown in obese women suffering from polycystic ovary syndrome. A total of 57 patients who were treated with liraglutide and genotyped for rs6923761 and rs10305420 were included in the study. Individuals who, following a treatment duration of 12 weeks, had shed at least 5% of their starting body weight were categorized as strong responders. While the GLP1R rs10305420 polymorphism was linked to inadequate treatment response, individuals with at least one polymorphic rs6923761 allele tended to experience greater weight loss. The reductions in fasting glucose levels and those stimulated by the oral glucose tolerance test were similar for both groups [142].

**Table 5:** Summary of genetic polymorphisms influencing GLP-1RA response in T2DM.

Study Focus	Genetic Polymorphisms Investigated	Findings
Glycemic Response and Insulin Secretion	rs3765467, rs10305420, rs6923761, rs10305492	- rs3765467 GG genotype associated with greater HbA1c reduction and treatment response in Chinese T2DM patients [22]. - rs10305420 T allele homozygosity linked to better glycemic response to liraglutide in Iranian T2DM patients [134]. - rs6923761 showed no association with HbA1c reduction [22]. - rs3765467 and rs10305492 linked to impaired insulin secretion and β cell apoptosis [135]. (The exact mechanism by which these two SNPs can influence insulin secretion following GLP-1RA treatment in T2DM still not established.)
Stress Response	rs1042044	- rs1042044 C allele linked to increased response bias in reward tasks. - AA genotype linked to higher anhedonia scores. - No significant association with depression diagnosis. (The exact mechanism by which this SNP can influence stress response following GLP-1RA treatment in T2DM still not established.)
Cardiovascular Risk Factors	rs6923761, rs10305420	- rs6923761 showed no impact on metabolic or cardiovascular risk factors in Spanish T2DM patients [137]. - rs4714210 GG genotype associated with lower cardiovascular disease risk in Chinese Han T2DM patients [139]. (The exact mechanism by which this SNP can influence cardiovascular risk factors following GLP-1RA treatment in T2DM still not established.)
Body Weight and Metabolic Parameters	rs6923761, rs10305420	- rs6923761 A allele linked to greater weight loss and fat mass reduction [140]. - rs6923761 minor allele associated with delayed gastric emptying but no significant weight loss effect after 5 weeks [141]. - rs10305420 linked to poor weight loss response to liraglutide in obese women with PCOS [142].

## 8. Conclusions

GLP-1 RAs have emerged as powerful and multifaceted agents in managing metabolic disorders such as diabetes and obesity, and their utility is still expanding into areas that were previously beyond the scope of glycemic control. Their hepatoprotective effects in reducing hepatic steatosis, renoprotective properties in diabetic kidney disease, and cardioprotective capabilities in reducing cardiovascular events demonstrate their wide therapeutic potential. Moreover, GLP-1 RAs have shown remarkable promise in neuropsychiatric and neurodegenerative domains suggesting intriguing new treatment interventions. The discovery of genetic polymorphisms influencing responses to GLP-1 RAs emphasizes the necessity for personalized medicine strategies to improve patient outcomes. However, further research is required to determine long-term safety, efficacy across diverse populations, and a better understanding of the mechanisms behind these broad benefits. As such, GLP-1 RAs stand as pivotal therapies not just for diabetes management but also as a critical treatment for interconnected metabolic and psychiatric disorders.

## Data Availability

No new data were created or analyzed in this study. Data sharing is not applicable to this article.
